# Amino Acid‐Starved Cancer Cells Utilize Macropinocytosis and Ubiquitin‐Proteasome System for Nutrient Acquisition

**DOI:** 10.1002/advs.202304791

**Published:** 2023-11-20

**Authors:** Tianyi Wang, Yaming Zhang, Yuwei Liu, Yi Huang, Weiping Wang

**Affiliations:** ^1^ State Key Laboratory of Pharmaceutical Biotechnology The University of Hong Kong Hong Kong China; ^2^ Department of Pharmacology and Pharmacy Li Ka Shing Faculty of Medicine The University of Hong Kong Hong Kong China; ^3^ Dr. Li Dak‐Sum Research Centre The University of Hong Kong Hong Kong China

**Keywords:** amino acid starvation, cancer starvation therapy, macropinocytosis, pH‐responsive polymersomes, ubiquitin‐proteasome system

## Abstract

To grow in nutrient‐deprived tumor microenvironment, cancer cells often internalize and degrade extracellular proteins to refuel intracellular amino acids. However, the nutrient acquisition routes reported by previous studies are mainly restricted in autophagy‐lysosomal pathway. It remains largely unknown if other protein degradation systems also contribute to the utilization of extracellular nutrients. Herein, it is demonstrated that under amino acid starvation, extracellular protein internalization through macropinocytosis and protein degradation through ubiquitin‐proteasome system are activated as a nutrient supply route, sensitizing cancer cells to proteasome inhibition. By inhibiting both macropinocytosis and ubiquitin‐proteasome system, an innovative approach to intensify amino acid starvation for cancer therapy is presented. To maximize therapeutic efficacy and minimize systemic side effects, a pH‐responsive polymersome nanocarrier is developed to deliver therapeutic agents specifically to tumor tissues. This nanoparticle system provides an approach to exacerbate amino acid starvation for cancer therapy, which represents a promising strategy for cancer treatment.

## Graphical abstract

Under amino acid starvation, macropinocytosis and ubiquitin‐proteasome system are activated as a nutrient supply route. Therefore, amino acid deprivation also sensitizes cancer cells to proteasome inhibition. By blocking macropinocytosis and proteasome simultaneously, an innovative approach is presented to exacerbate amino acid starvation in cancer therapy. A pH‐responsive polymersome nanocarrier is developed to maximize therapeutic efficacy and minimize systemic side effects.

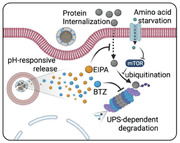

## 1 Introduction

As one of the leading causes of death worldwide, cancer is characterized by uncontrolled cell proliferation and highly progressive nature. Solid tumors often outstrip their blood supply and develop a tumor microenvironment deprived of nutrients, such as amino acids and glucoses.^[^ ^1^ ^]^ As the most abundant organic constituents in the body fluid, proteins have the potential to serve as an alternative resource as each protein can provide a large number of amino acids by several orders of magnitude.^[^ ^1b^ ^]^ Therefore, cancer cells develop strategies to utilize extracellular proteins. For example, under amino acid starvation, extracellular proteins are internalized through macropinocytosis.^[^ ^1^, ^2^ ^]^ Mammalian target of rapamycin (mTOR) signaling pathway is inhibited and mTOR complex 1 (mTORC1) is released from the lysosome membrane, leading to vacuolar‐type H^+^ ATPase (V‐ATPase) assembly at the lysosomes. As active proton pumps, these V‐ATPases decrease lysosomal pH to increase protease activity and promote lysosomal degradation of protein contents.^[^ ^3^ ^]^ Decreased mTOR activity also triggers Unc51‐like kinase 1/2 activation, enhancing autophagosome formation and autophagic protein degradation.^[^ ^1^, ^4^ ^]^ Yet, the nutrient acquisition pathways demonstrated in previous studies are mainly restricted in autophagy‐lysosomal system. It remains largely unknown if other protein degradation pathways also play important roles in the utilization of extracellular proteins.

As another primary intracellular protein degradation system, ubiquitin‐proteasome system (UPS) accounts for degradation of 80–90% proteins, including short‐lived, native, misfolded, or damaged proteins, in a highly selective manner.^[^ ^5^ ^]^ Initially, ubiquitin is activated by binding to the active site cysteine of the ubiquitin‐activating enzyme (E1). Subsequently, ubiquitin is transferred to the ubiquitin conjugase (E2) and ultimately conjugated to lysine residue or N‐terminal amino group of the substrates by the ubiquitin ligase (E3). Eventually, the protein with polyubiquitinated chains is recognized and degraded by the proteasome.^[^ ^6^ ^]^ UPS controls various basic cellular processes such as cell cycle progression, signal transduction, metabolism, protein quality control, and anti‐tumor immune response.^[^ ^7^ ^]^ However, it is not thoroughly understood whether UPS is activated by amino acid starvation for extracellular protein degradation to supply amino acid pool for cancer cell survival. Identifying the influence of amino acid starvation on UPS activity may provide a potential target for cancer starvation therapy, as proteasome inhibition may efficiently block nutrient supply in starvation‐adapted cancer cells with high UPS activity. Since UPS mediates intracellular protein degradation, proteins in the extracellular space need to be internalized before the proteolytic process. When proteasome inhibition blocks UPS‐dependent protein degradation, insufficient intracellular amino acids may trigger compensatory protein internalization, which may lead to resistance to the starvation therapy. Therefore, it is highly desired to both block protein internalization and degradation for cancer starvation therapy.

**Figure 1:**
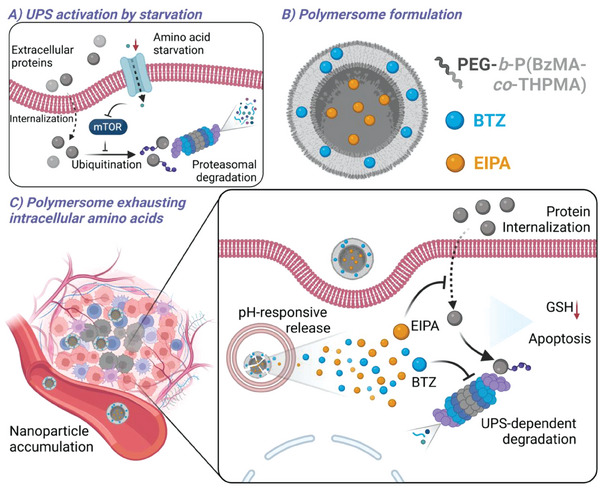
Schematic illustration depicting the pH‐responsive polymersomes loaded with BTZ and EIPA exhausting intracellular amino acids for cancer starvation therapy. Created with BioRender.com.

In this study, besides the traditional lysosomal pathway for extracellular protein utilization, we found macropinocytosis‐UPS axis can serve as another route for extracellular protein internalization and degradation. As a result, starved cancer cells became more sensitive to the UPS inhibition. Therefore, UPS may serve as a novel target for cancer starvation therapy. Our results showed that proteasome inhibition enhanced protein internalization as a feedback, which may provide supplementary nutrients through autophagic degradation. Based on the macropinocytosis‐UPS axis as the amino acid replenishment route, we hypothesized that concurrent inhibition of macropinocytosis and proteasome could significantly exacerbate amino acid starvation. Therefore, we explored the potential of the combination therapy with a UPS inhibitor, bortezomib (BTZ), and a macropinocytosis inhibitor, 5‐(n‐ethyl‐n‐isopropyl)‐amiloride (EIPA) in cancer treatment. To co‐deliver both hydrophobic drug, BTZ, and hydrophilic drug, EIPA, a pH‐responsive polymersome delivery system was developed to further enhance drug accumulation at tumor tissues and control drug release in cancer cells. In stark contrast with previous nanomedicines consuming glucose or blocking amino acid transporter, we, for the first time, developed a coordinative nanocarrier targeting protein catabolism, as a promising strategy for starvation‐based cancer therapy (**Figure** **1**).

## 2 Results

### 2.1 Amino Acid‐Mediated Starvation Activates UPS for Internalized Protein Degradation

**Figure 2:**
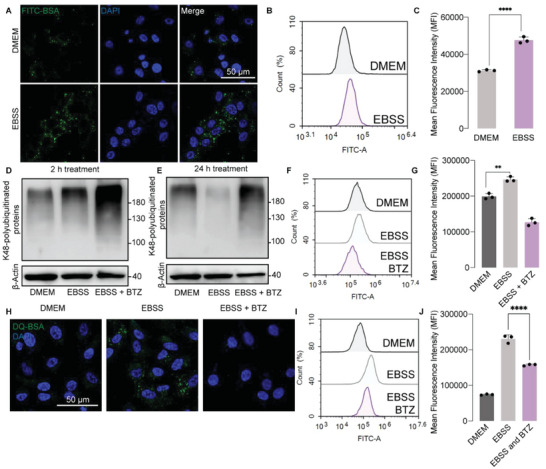
Amino acid‐mediated starvation induces UPS activation for internalized protein degradation. A) Representative CLSM images of FITC‐BSA (0.2 mg mL^−1^) internalized by A549 cancer cells treated with DMEM or EBSS medium for 4 h. B) Flow cytometry analysis, and C) quantified result of FITC‐BSA (0.2 mg mL^−1^) internalization in A549 cells treated with DMEM or EBSS medium for 4 h. D,E) Western blot analysis of K48‐polyubiquitinated proteins in A549 cells treated with DMEM, EBSS, EBSS plus BTZ (10 µm) for 2 h or 24 h. F) Flow cytometry result, and G) quantified analysis of proteasome activity experiment in A549 cells receiving DMEM, EBSS, EBSS plus BTZ (10 µm) for 4 h. H) Representative CLSM images of DQ‐BSA (10 µg mL^−1^) internalization and degradation analysis in A549 cells receiving DMEM, EBSS, EBSS plus BTZ (10 µm) for 4 h. I) Flow cytometry result, and J) quantified analysis of DQ‐BSA uptake and degradation analysis in A549 cells receiving indicated treatments for 4 h. Data are represented as mean ± SD (*n* = 3). ^**^ *p* <0.01, ^****^ *p* <0.0001.

First, we investigated the influence of amino acid‐mediated starvation on protein internalization. To mimic amino acid starvation environment, human lung adenocarcinoma epithelial cells (A549) were cultured in Earle's balanced salt solution (EBSS) medium as a starvation model.^[^ ^1^, ^8^ ^]^ Fluorescein isothiocyanate‐conjugated bovine serum albumin (FITC‐BSA) was employed as a fluorescent biomarker to trace protein internalization. Compared with cells cultured in a normal condition, starved cancer cells displayed higher FITC‐BSA internalization, demonstrated by confocal laser scanning microscopy (CLSM) (**Figure** **2**A) and flow cytometry analysis (Figure 2B,C). We further explored whether UPS could be activated in cancer cells subjected to amino acid starvation. For most proteins, ubiquitination is the rate‐limiting step for protein breakdown by UPS,^[^ ^9^ ^]^ we therefore evaluated the level of K48‐polyubiquitinated proteins after starvation, as K48‐linked polyubiquitin chains are primarily involved in proteasomal degradation.^[^ ^10^ ^]^ Interestingly, in stark contrast with the cells maintained in Dulbecco's modified Eagle's medium (DMEM), short‐term starvation (2 h) facilitated protein ubiquitination, which was notably decreased after long‐term treatment (24 h), implying that depletion of amino acids facilitated protein ubiquitination at the early stage and promoted degradation of these polyubiquitinated proteins at the late stage (Figure 2D,E). As a UPS inhibitor, BTZ led to profound accumulation of polyubiquitinated proteins both in short‐term and long‐term starved cancer cells, as it blocked proteasomal degradation (Figure 2D,E). All these results indicated that amino acid starvation promoted protein ubiquitination and proteasomal degradation.

To further measure the UPS activity, a fluorescent proteasome probe (Me4BodipyFL‐Ahx3Leu3VS) was utilized, which can specifically bind to the active site of proteasome and label it with green fluorescence. As indicated by flow cytometry, the fluorescence intensity of proteasome probe was elevated in amino acid‐starved cancer cells and remarkably reversed by BTZ treatment (Figure 2F,G). Moreover, a constitutively fluorescent albumin (DQ‐BSA) was applied to trace extracellular protein internalization and degradation process, which will only emit green fluorescence after albumin degradation. In starved cancer cells, the fluorescence intensity of DQ‐BSA was increased compared with cells in normal medium and substantially decreased by BTZ, indicating that starvation facilitated internalized protein degradation in a UPS‐dependent manner (Figure 2H–J).

**Figure 3:**
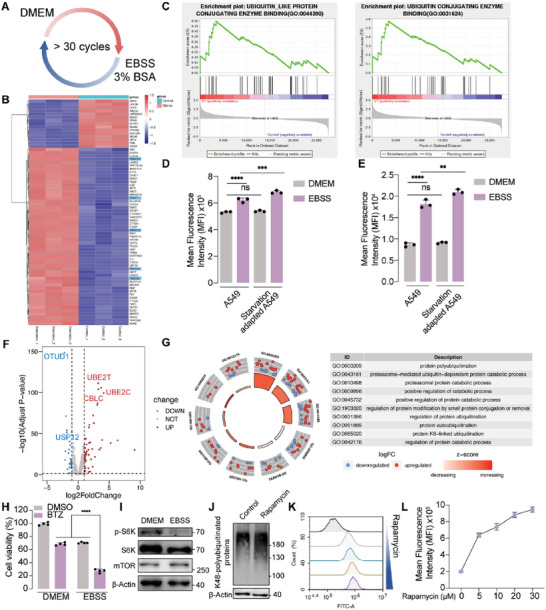
UPS activity in starvation‐adapted A549 cells and LUAD tumor tissues. A) Outline of the process for starvation‐adapted cell line construction. B) Top 70 DEGs of RNAseq analysis between normal A549 cells and starvation‐adapted A549 cells. C) GSEA analysis of all DEGs between normal A549 cells and starvation‐adapted A549 cells. D) Flow cytometry analysis of proteasome activity in normal A549 cells and starvation‐adapted A549 cells, treated with normal DMEM or EBSS, separately. E) Flow cytometry analysis of FITC‐BSA internalization in normal A549 cells and starvation‐adapted A549 cells, treated with normal DMEM or EBSS, separately. F) Volcano plot of UPS‐associated DEGs between lung tumor tissues and surrounding normal tissues. G) GO enrichment analysis of UPS‐associated DEGs between lung tumor tissues and surrounding normal tissues. H) Cell viability analysis of A549 cells treated with DMSO or BTZ (20 µM), in DMEM or EBSS medium for 24 h. I) Western blot analysis of p‐S6K (70 kDa), S6K (70 kDa), and mTOR (289 kDa) in A549 cells treated with normal DMEM or EBSS medium for 2 h. J) Western blot analysis of K48‐polyubiquitinated proteins in A549 cells treated with DMSO or rapamycin (100 µm) for 2 h. K) Flow cytometry and L) quantified analysis of proteasome activity probe in A549 cells treated with a series of concentrations of rapamycin for 4 h. Data are represented as mean ± SD (*n* = 3). ^**^ *p* <0.01, ^***^ p<0.001,^****^ *p* <0.0001, ns: not significant.

As solid tumors often develop into a chronic starvation microenvironment, we therefore investigated the UPS activity using a long‐term, starvation‐adapted cancer cell model.^[^ ^1a^ ^]^ The cells were cultured in normal DMEM and EBSS (with 3% BSA) periodically for more than 30 generations (**Figure** **3**A). RNA sequencing (RNAseq) analysis was applied, and differentially expressed gene (DEG) analysis was performed between normal and starvation‐adapted A549 cells. Among the top 70 DEGs, 5 UPS‐related genes, including TNFAIP3, TRAF1, ARRDC4, FBXW10, and FBXO32, were upregulated in the starvation group. And all these five genes were positively associated with higher UPS activity (Figure 3B). Gene set enrichment analysis (GSEA) was also investigated, suggesting that starvation was positively correlated with ubiquitin‐like protein conjugating enzyme binding and ubiquitin conjugating enzyme binding activity (Figure 3C). Intriguingly, when cultured in complete DMEM, starvation‐adapted cells exhibited similar level of proteasome activity (Figure 3D) and FITC‐BSA uptake (Figure 3E) compared with normal A549 cells. While under amino acid deprivation, starvation‐adapted cells demonstrated higher proteasome activity (Figure 3D) and FITC‐BSA internalization (Figure 3E).

Considering high UPS activity elicited by amino acid starvation, we hypothesize that the UPS remains highly active in tumor tissues, which is more likely to be subjected to low nutrient supply in comparison to normal tissues.^[^ ^11^ ^]^ Therefore, we collected clinical RNAseq data from the cancer genome atlas (TCGA) database and compared DEGs between lung adenocarcinoma (LUAD) and normal tissues. As a result, genes encoding ubiquitin conjugating enzymes or ligases, such as UBE2T, UBE2C, and CBLC, were up‐regulated in tumor tissues, while genes encoding deubiquitinating enzymes like OTUD1 and USP12 were down‐regulated (Figure 3F). Gene ontology (GO) enrichment analysis also implied that lung cancer harnessed activated UPS‐related pathways compared with normal tissues (Figure 3G). Moreover, univariate Cox regression analysis was also investigated to identify that 23 genes among these 77 DEGs were significantly associated with LUAD patients’ survival. And most of these genes were associated with poor prognosis in patients (Figure S1, Supporting Information). Using Lasso regression analysis, three genes including PRC1, TRIM6, and TRIML2 were found to act as independent prognostic signatures for patients. Higher expression of these three hallmark genes represented higher risk and lower survival rate in the patients (Figure S2, Supporting Information). As UPS activity was associated with prognosis in clinical patients, we investigated the anti‐cancer effect of BTZ, especially in starved cancer cells. As a result, BTZ prompted a much more significant decrease of cell viability in starved cancer cells, in contrast to cells maintained in normal culture medium (Figure 3H). To summarize, we found amino acid starvation induced UPS activation for extracellular protein degradation and rendered cells more vulnerable to UPS blockage. In clinical dataset, cancer lesions displayed up‐regulation of UPS‐promoting genes in contrast to normal tissues. And higher UPS‐promoting gene expression was associated with poor prognosis in LUAD patients.

### 2.2 Starvation‐Activated UPS is Mediated by mTOR Signaling Inhibition

mTOR signaling pathway is a critical node in coordinating protein and amino acid homeostasis. Sufficient intracellular amino acids activate mTORC1, which phosphorylates S6 kinase (S6K) and 4E binding protein (4E‐BP) to initiate 5′ cap‐dependent protein translation.^[^ ^12^ ^]^ Conversely, amino acid deprivation leads to mTORC1 inhibition and activates degradation of endocytosed proteins.^[^ ^1^, ^13^ ^]^ As mTORC1 activity constitutes a sensitive mechanism to monitor amino acids recovered from internalized proteins, we hypothesize that starvation‐induced UPS activation maybe associated with mTOR signaling. A549 cancer cells were cultured in DMEM or EBSS medium for 24 h and S6K phosphorylation was evaluated using western blot analysis. According to the result, EBSS treatment led to lower S6K phosphorylation compared with the DMEM‐treated group, indicating lower mTORC1 signaling activity (Figure 3I). Rapamycin was employed as a mTORC1 inhibitor to further demonstrate the influence of mTORC1 activity on UPS level. According to the western blot result, the level of polyubiquitinated proteins was elevated by rapamycin (Figure 3J). Flow cytometry analysis was also employed to identify that rapamycin treatment enhanced proteasome activity in a dose‐dependent manner (Figure 3K,L). These results indicated that amino acid starvation can decrease mTORC1 activity, which can promote protein ubiquitination and elevate proteasome activity.

### 2.3 Synergistic Effect of UPS Inhibitor and Macropinocytosis Inhibitor

**Figure 4:**
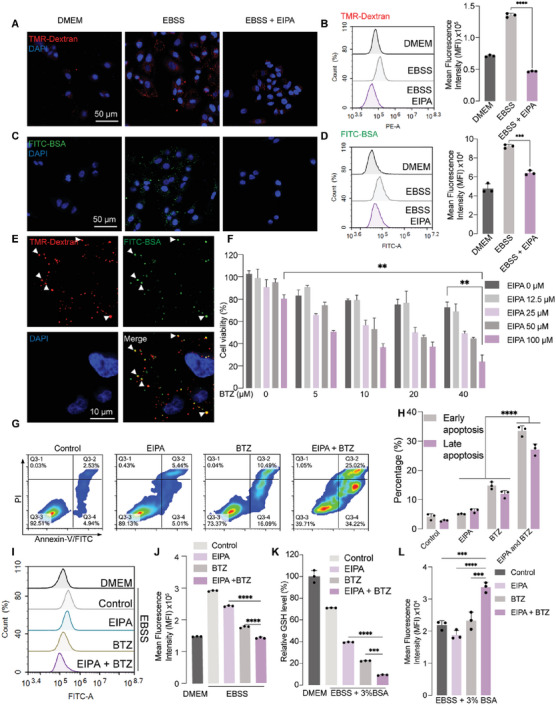
Synergistic anti‐cancer effect of EIPA and BTZ. A) Representative CLSM images of TMR‐Dextran internalization in A549 cells treated with DMEM, EBSS, or EBSS plus EIPA (50 µm, pretreated 1 h before) for 1 h. B) Flow cytometry and quantified analysis of TMR‐Dextran internalization in A549 cells treated with DMEM, EBSS, or EBSS plus EIPA (50 µm, pretreated 1 h before) for 1 h. C) Representative CLSM images of FITC‐BSA internalization in A549 cells treated with DMEM, EBSS, or EBSS plus EIPA (50 µM, pretreated 1 h before) for 1 h. D) Flow cytometry and quantified analysis of FITC‐BSA internalization in A549 cells treated with DMEM, EBSS, or EBSS plus EIPA (50 µM, pretreated 1 h before) for 1 h. E) Representative co‐localization analysis of FITC‐BSA and TMR‐Dextran in A549 cells using CLSM. F) Cell viability analysis of A549 cells treated with different concentrations of EIPA and BTZ for 24 h in EBSS medium. G) Flow cytometric apoptosis analysis, and H) quantified result of A549 cells treated with EIPA (50 µM), BTZ (20 µm), or their combination in EBSS medium for 24 h. I) Flow cytometry, and J) quantified analysis of DQ‐BSA fluorescence intensity in A549 cells treated with EIPA (50 µM), BTZ (20 µM), or their combination in DMEM or EBSS medium for 24 h. K) The level of GSH in A549 cells treated with EIPA (50 µM), BTZ (10 µm), or their combination in DMEM or EBSS (3% BSA) medium for 24 h. L) DCFH‐DA flow cytometric analysis in A549 cells treated with EIPA (50 µm), BTZ (10 µm), or their combination in indicated medium for 24 h. Data are represented as mean ± SD (n = 3). ^**^p <0.01, ^***^ *p* <0.001, ^****^ *p* <0.0001.

Based on the results mentioned above, BTZ has the ability to block UPS‐dependent degradation of proteins in starved cancer cells, thereby limiting cell survival. However, extracellular proteins can still be internalized and degraded through lysosome‐dependent degradation pathways.^[^ ^14^ ^]^ Hence, the use of BTZ as a monotherapy for solid tumors may have limited anti‐cancer effects.^[^ ^15^ ^]^ Therefore, we hypothesize that simultaneously blocking protein internalization and UPS‐dependent degradation may further enhance the cancer starvation therapy. We focused on macropinocytosis pathway, because it plays an essential role in mediating protein internalization.^[^ ^2^ ^]^ Tetramethylrhodamine (TMR)‐Dextran was utilized as a marker to validate that macropinocytosis was significantly enhanced by amino acid starvation and inhibited by EIPA (**Figure** **4**A,B). Through the CLSM analysis, the co‐localization of FITC‐BSA and TMR‐Dextran was observed, suggesting that proteins can be internalized by macropinocytosis (Figure 4E). Presumably, the starvation‐amplified FITC‐BSA uptake was notably blocked by EIPA treatment (Figure 4C,D).

When we combined gradient concentrations of EIPA and BTZ together, the dual‐drug strategy prompted a notable decrease of cell viability compared with each single treatment (Figure 4F). The mean synergy scores calculated by SynergyFinder were higher than 20, implying that the cooperation of these two drugs was highly synergistic (Figure S3, Supporting Information).^[^ ^16^ ^]^ According to the apoptosis analysis, the dual‐drug strategy induced both higher early apoptosis (33.5%) and late apoptosis (27.1%) in contrast to each single treatment (Figure 4G,H). The combination also facilitated notable decrease of DQ‐BSA fluorescence intensity, attributed to the inhibition of protein internalization and UPS‐dependent degradation (Figure 4I,J). As amino acids, such as glutamate, cysteine and glycine, are requisite for glutathione (GSH) synthesis, the level of intracellular GSH may decrease as a result of reduced intracellular amino acid pool. Accordingly, the combination treatment induced a profound decrease of GSH (Figure 4K), representing a liability to oxidative stress. The dual‐drug combination led to the highest ROS generation evidenced by elevated 2′, 7′ ‐dichlorofluorescein diacetate (DCFH‐DA) fluorescence intensity compared with other groups (Figure 4L). Therefore, the decreased GSH level and elevated ROS generation may contribute to apoptotic cell death of the cancer cells.

### 2.4 Dual‐Drug Combination Avoids Compensatory Amino Acid Supply

**Figure 5:**
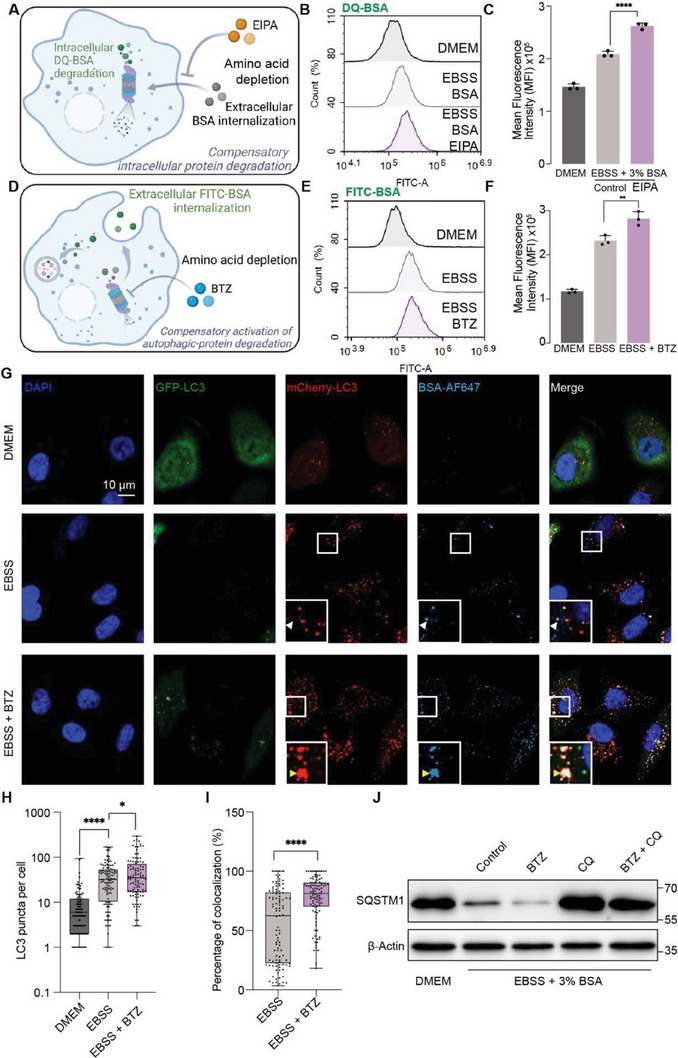
Combination of EIPA and BTZ circumvents compensatory protein catabolism. A) Scheme depicting the influence of EIPA on intracellular protein degradation. Created with BioRender.com. B) Flow cytometry and C) quantified analysis of cells pretreated with DQ‐BSA for 1 h, then cultured in DMEM, EBSS plus BSA, EBSS plus BSA and EIPA for another 3 h. D) Scheme depicting the effect of BTZ on extracellular protein internalization and lysosome‐dependent degradation. Created with BioRender.com. E) Floy cytometry and F) quantified analysis of FITC‐BSA internalization in A549 cells receiving DMEM, EBSS, or EBSS plus BTZ for 4 h. G) Representative CLSM images and H,I) quantified analysis of mCherry‐GFP‐LC3 reporter system and BSA‐AF647 internalization in A549 cells treated with DMEM, EBSS, and EBSS plus BTZ for 4 h using FIJI‐ImageJ software with ComDet plugin. J) Western blot analysis of SQSTM1 (62 kDa) in A549 cells treated with indicated drugs in DMEM or EBSS plus BSA medium for 4 h. Data are represented as mean ± SD (*n* = 3). ^*^ *p* <0.05, ^**^ *p* <0.01, ^****^ *p* <0.0001.

Besides phenotypically demonstrating the anti‐cancer effect, we also investigated the detailed mechanism of the synergistic effect. We proposed that although EIPA as a single agent can inhibit extracellular protein internalization in amino acid‐starved cancer cells, intracellular proteins may also be degraded to provide amino acids for cell survival. To define the influence of EIPA on intracellular protein degradation, we pre‐incubated the cells with DQ‐BSA for 1 h and changed the medium to DMEM, EBSS with 3% BSA, and EBSS with 3% BSA plus EIPA, and incubated for another 3 h, respectively. Hence, the change of fluorescence intensity was induced by different treatments after DQ‐BSA internalization, as a surrogate for intracellular protein degradation (**Figure** **5**A). According to flow cytometry analysis, EIPA further increased the fluorescence intensity of DQ‐BSA compared with starvation group, suggesting that EIPA led to compensatory degradation of intracellular DQ‐BSA through inhibiting unconjugated BSA uptake (Figure 5B,C).

In addition, single BTZ treatment may not influence protein uptake, hence extracellular proteins may still be internalized and degraded through lysosome‐dependent pathways (Figure 5D). When the cells were incubated with FITC‐BSA, treated with or without BTZ in DMEM or EBSS medium for 4 h, we observed that BTZ further increased protein internalization (Figure 5E,F). We transduced A549 cancer cells with GFP‐mCherry‐LC3 plasmid to define the autophagy flux after different treatments. According to the CLSM analysis, we observed a notable increase of LC3 puncta in starved cancer cells compared with the control group, which was further enhanced by BTZ incubation (Figure 5G,H). Moreover, compared with starved cancer cells, BTZ treatment increased the percentage of BSA‐AF647 colocalized with LC3 puncta, implying that BTZ drove compensatory extracellular protein uptake and autophagy‐dependent degradation (Figure 5G,I). As a chaperone protein for autophagy, SQSTM1 expression level was also investigated using western blot analysis. BTZ exacerbated starvation‐induced SQSTM1 decrease, indicating that autophagy was amplified for protein degradation, which was further validated by turnover assay using chloroquine (CQ) as a late‐autophagy inhibitor (Figure 5J). To summarize, single treatment by EIPA or BTZ may contribute to activation of compensatory amino acid supply route to benefit cancer cell survival. Therefore, blocking protein internalization and UPS‐dependent degradation simultaneously may provide a tentative strategy for cancer starvation therapy.

### 2.5 pH‐Responsive Polymersome as the Dual‐Drug Delivery System

**Figure 6:**
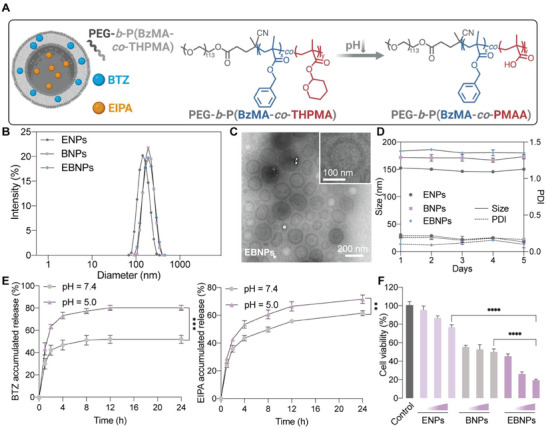
Characterization of polymersomes encapsulating EIPA and BTZ. A) Scheme of the polymersomes encapsulating EIPA and BTZ, which can undergo pH‐responsive cleavage. Created with BioRender.com. B) Average sizes of ENPs, BNPs, and EBNPs using DLS. C) TEM image of EBNPs. D) Stability test of ENPs, BNPs, and EBNPs in DMEM with 10% FBS at 37 °C for 5 days. E) Drug release profiles of EBNPs for 24 h at pH 7.4 and pH 5.0, separately. F) Cell viability of A549 cells treated with ENPs, BNPs, and EBNPs for 24 h. Concentrations of EIPA was 25, 50, or 100 µm. Concentrations of BTZ was 14, 28, or 56 µm. Data are represented as mean ± SD (*n* = 3). ^**^ *p* <0.01, ^***^ *p* <0.001, ^****^ *p* <0.0001.

To further facilitate the clinical translation of this combination approach, nanodrug delivery strategy was applied in this study to enhance the drug accumulation at tumor sites while reducing unwanted adverse effects through enhanced permeability and retention (EPR) effect.^[^ ^17^ ^]^ A novel polymersome was developed to co‐encapsulate EIPA into the hydrophilic core and BTZ in the hydrophobic membrane bilayer (**Figure** **6**A). It is noteworthy that a pH‐responsive tetrahydropyranyl methacrylate (THPMA) moiety was incorporated to facilitate drug release in acidic environment, allowing specific drug release in the tumor tissues.^[^ ^18^ ^]^ The synthetic routes of the block copolymers were demonstrated in Figure S4 (Supporting Information). The pH‐responsive monomer, THPMA, was synthesized using methacrylic acid and dihydropyran (Figure S5, Supporting Information). The block copolymer, PEG‐*b*‐P(BzMA‐*co*‐THPMA), was synthesized via the reversible addition‐fragmentation chain transfer (RAFT) copolymerization of BzMA and THPMA monomers using the PEG‐RAFT agent. The BzMA and THPMA monomer polymerization degrees were determined to be 68 and 63, as confirmed by ^1^H‐NMR analysis (Figure S6, Supporting Information). Hence, the synthesized block copolymer was referred as PEG_113_‐*b*‐P(BzMA_68_‐*co*‐THPMA_63_).

Subsequently, the copolymer was employed to form polymersomes to encapsulate EIPA and BTZ (Figure 6A). According to the dynamic light scattering (DLS) measurement, the sizes of EIPA‐loaded nanoparticles (ENPs), BTZ‐loaded nanoparticles (BNPs), and EIPA and BTZ‐loaded nanoparticles (EBNPs) were 143.5 nm, 188.9 nm, and 186.3 nm, respectively. And the polymer dispersity index (PDI) was all lower than 0.1 (Figure 6B,C). For the EBNPs, the encapsulation efficiency of EIPA and BTZ was 87.9 ± 3.46% and 68.87 ± 15.53%, respectively. The loading capacity of EIPA and BTZ was 35.37 ± 0.92% and 26.67 ± 4.11%, respectively. These nanoparticle formulations remained stable in DMEM with 10% FBS at 37 °C for at least 5 days (Figure 6D). ^1^H‐NMR analysis was employed to characterize the pH responsiveness of this block copolymer (Figure S7, Supporting Information), demonstrating that the THPMA moiety can be cleaved in acidic environment. Drug release profiles of the nanoparticles were characterized under pH 7.4 and pH 5.0, separately, using reversed‐phase high‐performance liquid chromatography (RP‐HPLC). According to the result, the release of BTZ and EIPA was increased under acidic environment and reached 80.2% and 71.5% at 24 h, respectively, suggesting that these polymersomes may rapidly release drugs in acidic environment (Figure 6E). We also incubated cancer cells with different formulations with gradient concentrations of drugs and validated that dual‐drug nanoparticles exhibited the highest cell killing effect in vitro (Figure 6F). And cell viability did not decrease significantly in HUVEC cells treated with different concentrations of polymersomes, suggesting a good biocompatibility of the polymeric materials (Figure S8, Supporting Information).

### 2.6 Anti‐Cancer Effect of Dual‐Drug Nanoparticles In Vivo

**Figure 7:**
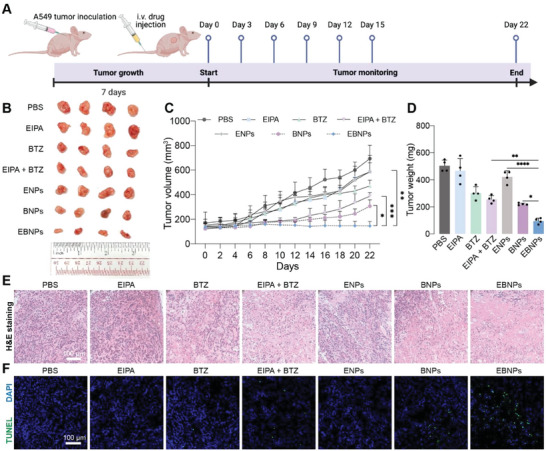
Anti‐tumor effect of polymersome nanoparticles in xenograft tumor mouse model. A) Schematic illustration of the schedule for xenograft tumor model implantation and synergistic therapy. B) Images of xenograft tumor tissues excised from tumor‐bearing mice at the endpoint of treatments (n = 4). C) Tumor volume profile of tumor‐bearing mice receiving different treatments. D) Tumor weight of xenograft tumor tissues excised from tumor‐bearing mice at the endpoint of treatments (n = 4). E) Representative H&E staining analysis of tumor tissues excised from tumor‐bearing mice at the endpoint of treatments. F) Representative TUNEL assay of tumor tissues excised from tumor‐bearing mice at the endpoint of treatments.^*^ *p*<0.05, ^**^ *p*<0.01, ^***^ *p*<0.001, ^****^ *p*<0.0001.

The applicability of the dual‐drug polymersomes in vivo was also evaluated using xenograft mouse model. A549 cancer cells were inoculated at the right shoulder of the BALB/c nude mice. The tumor‐bearing mice were divided into 7 groups, including PBS, EIPA, BTZ, EIPA + BTZ, ENPs, BNPs, and EBNPs. Different formulations (EIPA: 3 mg kg^−1^ and BTZ: 0.75 mg kg^−1^) were intravenously injected into the mice six times at an interval of three days, separately (**Figure** **7**A). As dual‐drug polymersome formulation, EBNPs exhibited significantly lower tumor volume and tumor weight compared with BNPs or ENPs (Figure 7B–D), due to the combination effect of these two molecules. EBNPs also prompted higher anti‐cancer effect in contrast to free‐drug combination, possibly due to higher drug retention in the tumor tissues as a nanoparticle formulation. Hematoxylin and eosin (H&E) staining analysis was performed in tumor tissues. In contrast to other groups, EBNPs treatment notably led to nuclear shrinkage and fragmentation with disappeared cell contour, suggesting desired ability to ablate tumor tissues (Figure 7E). Terminal deoxynucleotidyl transferase dUTP nick end labeling (TUNEL) assay was also performed to evaluate the cell apoptosis in tumor tissues. It was observed that EBNPs led to the highest percentage of apoptotic cells in tumor tissues, in contrast to other groups (Figure 7F). Biosafety profile was investigated to evaluate the biocompatibility of different formulations. Along the treatment procedure, all these groups did not show notable fluctuation of the body weight (Figure S9, Supporting Information). And there was no significant structural and morphological change in H&E staining of major organs including heart, liver, spleen, lung, and kidney (Figure S10, Supporting Information), suggesting a desired biocompatibility of injected formulations used in this study.

## 3 Discussion

Nutrient deprivation occurs in early stages of tumor development before formation of new blood vessels or in late stages owing to abnormal tumor vasculature.^[^ ^1a,c^ ^]^ Although this metabolic stress may restrict tumor growth, metabolically adapted cells could be selected due to chronic starvation, which may promote tumor progression.^[^ ^19^ ^]^ In starved cancer cells, extracellular proteins are engulfed through macropinocytosis, degraded through lysosome‐dependent pathway, and served as alternative resources to fuel amino acid pool.^[^ ^1^, ^20^ ^]^ As the other protein degradation pathway, UPS was traditionally assumed to serve distinct function with autophagy‐lysosome system.^[^ ^14a^ ^]^ Recent studies reported that starvation may also impact the UPS, while the results remained controversial. It was reported by Zhao et al. that amino acid starvation may enhance proteasome activity in HEK293 cells dependent on mTOR inhibition.^[^ ^14a^ ^]^ While starvation may also elicit polyubiquitination of 26S proteasome and traffic it into autophagosome for autophagy‐dependent degradation.^[^ ^21^ ^]^

In this study, we defined that amino acid deprivation prompted high proteasome activity (Figure 2F,G), thereby leading to augmented UPS‐dependent internalized protein degradation in cancer cells (Figure 2H–J). A starvation‐adapted cancer cell line was constructed to mimic chronically starved tumor microenvironment.^[^ ^8^ ^]^ Interestingly, this cell line adapted to long‐term metabolic stress preferentially utilized amino acids in complete medium, while exhibited higher ability to internalize extracellular proteins for degradation under amino acid deprivation (Figure 3D,E). According to RNAseq analysis, the starvation‐adapted cell line was characterized by up‐regulation of UPS‐promoting genes and activation of ubiquitination process (Figure 3A–C). In addition, based on clinical TCGA database, the UPS‐related genes and pathways were also positively enriched in tumor tissues compared with normal tissues (Figure 3F,G), associated with poor prognosis in LUAD patients (Figures S1,S2, Supporting Information). As starvation sensitized cancer cells to BTZ (Figure 3H), UPS represented a potential target for cancer starvation therapy.

Before polyubiquitination and proteasomal degradation processe, extracellular proteins need to be internalized in cancer cells. It was observed in previous studies that macropinocytosis played a critical role in mediating extracellular protein uptake, which can be inhibited by macropinocytosis inhibitor, such as EIPA.^[^ ^2^ ^]^ And necrotic cell debris can also be internalized through macropinocytosis and provide nutrients for cell survival and rendered cancer cells resistant to chemotherapy or radiotherapy.^[^ ^22^ ^]^ Therefore, given that macropinocytosis and proteasomal degradation could be an amino acid supply route, we hypothesize that blocking macropinocytosis and UPS‐dependent degradation at the same time may exacerbate nutrient deprivation. As expected, combination of EIPA and BTZ displayed a synergistic cytotoxicity through promoting ROS generation (Figure 4L) and apoptotic cell death (Figure 4F–H). This dual‐drug strategy also decreased intracellular GSH level dramatically, demonstrating liability to oxidative stress (Figure 4K). Intriguingly, while EIPA treatment alone can inhibit internalization of proteins (Figure 4C,D), it also resulted in compensatory intracellular protein degradation (Figure 5A–C). Moreover, BTZ single treatment stimulated compensatory protein internalization, which can refuel amino acids by autophagic degradation (Figure 5D–J).^[^ ^23^ ^]^ Through bypassing these mechanisms, the dual‐drug combination strategy exhausted intracellular amino acids and prompted an exacerbated anti‐cancer effect.

While these two small molecules were applied as a combination strategy to eliminate cancer cells, the clinical implementation could be further promoted by a nanodrug delivery system to co‐deliver these two drugs, simplifying clinical administration.^[^ ^24^ ^]^ With prolonged circulation time, nanodrugs preferentially accumulate into tumor tissues due to permeable tumor vasculature and defective lymphatic drainage, known as EPR effect.^[^ ^25^ ^]^ And water solubility of EIPA and BTZ can also be increased to elevate effective concentration in blood circulation.^[^ ^26^ ^]^ Therefore, a polymersome nanocarrier was developed to co‐encapsulate these two drugs. It is noteworthy that previous nanoparticle‐based approaches for cancer starvation therapy primarily focus on glucose consumption or amino acid transporter blockage, while we for the first time developed a coordination system targeting protein catabolism.^[^ ^27^ ^]^ According to the results, the pH‐responsive THPMA moiety facilitated drug release in acidic environment (Figure 6D).^[^ ^18a^ ^]^ The dual‐drug polymersomes (EBNPs) exerted higher anti‐cancer effect compared with free‐drug administration or single‐drug polymersomes (ENPs or BNPs) (Figure 7B–D). EBNPs also displayed significantly higher ability to ablate the tumors and elicit apoptosis in tumor tissues (Figure 7E,F).

## 4 Conclusion

In conclusion, besides the conventional lysosomal pathway for extracellular nutrient utilization, we found macropinocytosis‐UPS axis can serve as another extracellular protein degradation process for amino acid supply to support cancer cell survival. Starved cancer cells displayed higher sensitivity to BTZ. Therefore, we combined BTZ with macropinocytosis inhibitor, EIPA, to block protein internalization and UPS‐dependent degradation at the same time, to achieve synergistic anti‐cancer effect by avoiding compensatory protein catabolism. To facilitate the clinical translation of this strategy, a pH‐responsive polymersome was developed to co‐deliver these two drugs, allowing enhanced drug retention in tumor tissues and higher therapeutic efficacy. By targeting protein catabolic process, this study provides a novel insight into cancer starvation therapy.

## 5 Experimental Section

### Materials

EIPA hydrochloride and BTZ were purchased from MedChemExpress LLC (New Jersey, USA). The proteasome probe, Me4BodipyFL‐Ahx3Leu3VS, was purchased from R&D system (Minnesota, USA). Dimethyl sulfoxide (DMSO) was obtained from Sigma‐Aldrich (Darmstadt, Germany). 3‐(4,5‐dimethyl‐2‐thiazolyl)‐ 2,5‐diphenyl‐2‐H‐tetrazolium bromide (MTT) was obtained from J&K Co., Ltd (Beijing, China).

### Cell Culture

A549, HUVEC and 293 T cell lines were purchased from ATCC and used in this study. High glucose (4.5 g/mL) Dulbecco modified Eagle medium (DMEM, Gibco) containing 10% fetal bovine serum (FBS, Gibco) and 1% penicillin/streptomycin (Gibco) were used for cell culture. The cells were incubated in the incubator under 100% humidity, 5% CO_2_, 37°C condition. For amino acid starvation, the cells were cultured in Earle's balanced salt solution (EBSS, Gibco), supplemented with glucose solution (Gibco) and MEM Vitamin solution (Gibco). To construct the starvation‐adapted A549 cell line, the cells were incubated with EBSS (with 3% BSA) and complete DMEM periodically. The cells adapted to starvation were successfully constructed after 30 generations.

### Fluorescent Imaging Analysis

For in vitro fluorescent imaging detection, the cells were seeded on Nunc glass bottom confocal dishes (Thermo Fischer Scientific, USA) for 24 h. Unless explained, the cells were incubated with FITC‐BSA (0.2 mg mL^−1^), BSA‐AF647 (0.05 mg mL^−1^), or DQ‐BSA (10 µg mL^−1^) for 4 h for protein internalization and extracellular protein degradation analysis, respectively. TMR‐Dextran (0.125 mg mL^−1^) was applied for 1 h as a macropinocytosis marker. Subsequently, after washed with PBS three times, the cells were fixed in 3.7% paraformaldehyde solution in PBS for 10 min, followed by washing with PBS three times and staining with DAPI according to the protocol (Thermo Fischer Scientific, D1306). After washing with PBS three times, the cells were mounted with SlowFade Diamond Antifade Mountant (Thermo Fischer Scientific, S36963). The in vivo TUNEL assay was conducted using the one‐step TUNEL assay kit (C1088, Beyotime, China). The Zeiss LSM 900 inverted confocal microscope equipped with 10 × 0.45 NA, 20 × 0.8 NA and 40 × 1.4 NA lens was employed to analyze intracellular fluorescent signals. The same exposure settings were used across all conditions in each individual experiment.

### Flow Cytometry Analysis

The cells were seeded in 12‐well plates at 1 × 10^5^ density. Unless explained, the cells were incubated with FITC‐BSA (0.2 mg/mL), DQ‐BSA (10 µg mL^−1^), or Me4BodipyFL‐Ahx3Leu3VS (1 µm) for 4 h for protein internalization, extracellular protein degradation, and proteasome activity analysis, respectively. TMR‐Dextran (0.125 mg mL^−1^) was applied for 1 h as a macropinocytosis marker. After washing with PBS three times, the cells were harvested and analyzed by Agilent NovoCyte Quanteon analyzer.

### Western Blot

The cells were plated in 12‐well plates at 1 × 10^5^ density and incubated for 24 h. Subsequently, the cells received indicated treatments for 4 h and the cells were washed with 1x PBS three times and harvested with RIPA buffer (supplemented with Halt protease and phosphatase inhibitor cocktail, Thermo Fischer Scientific, USA). Total protein concentration was determined by pierce BCA protein assay kit (Thermo Fischer Scientific, #23225). Equal amounts of proteins were loaded. SDS‐PAGE electrophoresis was utilized to separate proteins, which were subsequently transferred to a nitrocellulose membrane through the wet transfer system. The membranes were blocked with 5% BSA in TBST at room temperature for 1 h and incubated with indicated primary antibodies overnight under 4 °C. After washed with TBST, the blots were incubated with secondary antibodies for 1 h at room temperature. After washed with TBST three times, the proteins blots were visualized by Clarity Western ECL Substrate (BioRad) and captured by ChemiDoc Imaging System (BioRad). The following antibodies were used: K48‐polyubiquitinated proteins (Cell signaling technology, #8081), p‐S6K (Cell signaling technology, #9234), S6K (Cell signaling technology, #2708), mTOR (Cell signaling technology, #2983), β‐Actin (Cell signaling technology, #4967), SQSTM1(Abcam, ab91526), HRP‐conjugated secondary antibody (Abcam, ab6789), HRP‐conjugated secondary antibody (Abcam, ab6721).

### Cytotoxicity Assay

The cells were seeded in 96‐well plates and cultured for 24 h. To investigate the influence of starvation on BTZ treatment, the cells were incubated with BTZ (20 µM) or DMSO, in complete DMEM or EBSS medium for 24 h. To investigate the combination effect of EIPA and BTZ, the cells were treated with a series of concentration of EIPA and BTZ in EBSS (plus 3% BSA) medium for 24 h. 10 µL of MTT (5 mg mL^−1^) were added into each well and incubated for 2–4 h. Then the supernatant was changed with 100 µL DMSO to dissolve the precipitate. Subsequently, the absorbance of the solution at 570 and 630 nm was measured by a microplate reader (SpectraMax M4, Molecular Devices LLC, San Jose, CA). The cell viability was calculated by OD570 – OD630 and normalized to DMSO control group.

### Apoptosis Analysis

The cells were plated in 12‐well plates at 1 × 10^5^ density and incubated for 24 h. Subsequently, the cells were incubated with EIPA (50 µm) or BTZ (20 µm), in EBSS (plus 3% BSA) medium for 24 h. Then the cells were washed with PBS three times, collected, and stained with Annexin‐V/PI double staining kit (Beyotime, C1062S) according to protocol. Then the apoptosis was analyzed by Agilent NovoCyte Quanteon machine.

### ROS Generation Analysis

The cells were seeded in 12‐well plates at 1 × 10^5^ density for 24 h and treated with DMSO or indicated drugs in EBSS medium with 3% BSA for 24 h. Subsequently, the cells were incubated with DCFH‐DA probe (10 µm) for 30 min. Then the cells were washed with PBS three times and harvested for flow cytometry analysis and the mean fluorescence intensity of DCFH‐DA was measured by Agilent NovoCyte Quanteon system.

### Glutathione (GSH) Level Analysis

The cells were seeded in 6‐well plates at 1.5 × 10^5^ density for 24 h and treated with DMSO or indicated drugs in EBSS medium with 3% BSA for 24 h. Then the cells were washed with 1x PBS and harvested for detection. Intracellular GSH level was analyzed according to the protocol of Glutathione Colorimetric Detection Kit (Invitrogen, EIAGSHC). Subsequently, the absorbance of the solution at 405 nm was measured by the microplate reader.

### mCherry‐GFP‐LC3 Transduction

The plasmid, pCDH‐CMV‐mC‐G‐LC3B‐P, was a gift from Kazuhiro Oka (Addgeneplasmid#124 974;http://n2t.net/addgene:124974;RRID:Addgene_124974). psPAX2 was a gift from Didier Trono (Addgene plasmid#12 260;http://n2t.net/addgene:12260;RRID: Addgene_12 260). pMD2.G was a gift from Didier Trono (Addgene plasmid#12 259;http://n2t.net/addgene:12259; RRID: Addgene_12 259). The mCherry‐GFP‐LC3 transduced A549 was constructed according to the previous publication.^[^ ^28^ ^]^ Generally, the mCherry‐GFP‐LC3 lentivirus was produced by transduction in 293T cells using lipofectamine 2000 (Invitrogen, USA) with helper plasmids psPAX2 and pMD2.G. After transduction (16 h), the cell culture medium was changed to complete DMEM medium. The supernatant was collected at 48 and 72 h after transduction, filtered through a 0.45 µm low‐binding filter, and frozen at −80 °C until use. For lentiviral transduction, A549 cells were seeded at 60–70% density and incubated with the mCherry‐GFP‐LC3 lentivirus supernatant with polybrene. Positive cells were selected using 1 µg mL^−1^ puromycin (GoldBio) for 2 weeks.

### Synthesis of Block Copolymer

The synthetic routes of pH‐responsive copolymer was depicted in Figure S4 (Supporting Information). First, the monomer, 2‐tetrahydropyranyl methacrylate (THPMA), was synthesized by acid‐catalyzed esterification of Methacrylic acid with dihydropyran. Briefly, Methacrylic acid (4.0 g, 41.5 mmol), p‐toluenesulfonic acid (0.35 g and 1.85 mmol) and pyridine (0.1467 g, 1.85 mmol) was dissolved in 35 mL of anhydrous dichloromethane. Dihydropyran (6.815 g and 81 mmol) was added into above solution dropwise, followed by stirring 24 h at room temperature. Subsequently, the reaction was terminated, and the solution was diluted with ethyl acetate and washed by brine. Finally, the organic solvent was collected and purified by column chromatography using ethyl acetate and hexane (1:6, v/v) as eluent to give pure THPMA monomer (5.15 g, yield: 66%).

Next, the amphiphilic pH‐responsive copolymer was synthesized via RAFT copolymerization of BzMA and THPMA monomers using PEG‐RAFT agent. Typically, PEG‐RAFT agent (26 mg, 0.005 mmol), 2,2′‐azobis(2‐methylpropionitrile) (0.115 mg, 0.0007 mmol), BzMA monomer (71 mg, 0.4 mmol), THPMA monomer (68 mg, 0.4 mmol), and 1,4‐dioxane (1 mL) were charged into a 5 mL Schlenk flask, evacuated by three freeze‐pump‐thaw cycles, and sealed under vacuum. The flask was placed in oil bath at 75 °C and the reaction was allowed for 18 h. Then the reaction solution was immersed into liquid nitrogen to terminate the reaction and added dropwise into cold diethyl ether for precipitation. Then the precipitate was centrifuged and collected. After repeating the dissolution‐precipitation process twice, the product was dried under vacuum to yield the white product (77 mg).

### Nanoparticle Preparation and Characterization

For the preparation of polysome nanoparticles, 2 mg polymers and 2 mg BTZ were dissolved in 1 mL organic solution (THF:DMSO = 4:1, v/v). And 2 mg EIPA hydrochloride was dissolved in 1 mL ddH_2_O. The EIPA solution was added into the stirring organic solution dropwise in 1 h using syringe pump (Longer Precision Pump Co., Ltd., China). Then 3 mL ddH_2_O was added into the stirring solution in 3 h. Unencapsulated drugs were removed by ultrafiltration. The size and polydispersity index of the nanoparticles were measured by dynamic light scattering instrument (ZS90, Malven Instrument, Southborough, MA, USA). The nanoparticles were also dissolved in DMEM (with 10% FBS) for stability test for 5 days at 37 °C.

### Drug Release Profile

The drug release profile of EBNPs under different pH value (7.4 and 5.0) at 37 °C was determined by dialysis method. 500 µL of EBNPs were dialyzed in a 3500 Da‐cutoff dialysis bag against 4 mL of PBS (pH 7.4 and pH 5.0). The outer solution was completely replaced at each time and fresh PBS was subsequently added. The cumulative release percentages of EIPA and BTZ over the time wereas calculated by HPLC measurement.

### Animal Studies

Female BALB/c nude mice (4–6 weeks) were purchased from Centre for Comparative Medicine Research (Li Ka Shing Faculty of Medicine, The University of Hong Kong). All animals received care and the experiments were based on the protocol approved by the Committee on the Use of Live Animals in Teaching and Research (CULATR) at Li Ka Shing Faculty of Medicine (CULATR No. 22–200). Animals were maintained at the conventional experimental holding area Dexter H.C. Man Building at the Centre for Comparative Medicine Research. To construct A549 xenograft breast cancer model, 3 × 10^6^ of A549 cells in DMEM, supplemented with Matrigel (Corning, 354 248) and collagen I (Gibco, A1048301) were subcutaneously implanted in the right flank of the mice. The treatment procedure was initiated when tumor volumes reached ≈100 mm^3^.

### Anti‐Tumor Efficacy Study

The tumor‐bearing mice were divided into seven groups (n = 4 per group). These seven groups of mice were treated with EIPA, BTZ, EIPA + BTZ, ENP, BNP, and EBNP. The concentration of EIPA and BTZ in different formulations were 3 and 0.75 mg kg^−1^, respectively. Different formulations were intravenously injected into the mice six times at an interval of 3 days. The body weight and tumor size of mice were measured every 2 days. Finally, after a 22‐day treatment, the mice were euthanized to isolate the tumor tissues for further analysis.

### H&E Staining

The tumor tissues and major organs (heart, liver, spleen, lung, and kidney) of these mice were harvested and H&E staining was performed to evaluate the anti‐cancer effect and biosafety profile, respectively. Briefly, 4 mm paraffin sections were dried under 60 °C for 2 h, dewaxed in xylene and rehydrated in gradient concentrations of ethanol step by step. The slides were stained with Mayers Hematoxylin for 1 min, washed in running tap water, acid ethanol and deionized water, sequentially. Then the slides were stained with Alcoholic‐Eosin for 1 min. Subsequently, the slides were dehydrated and rinsed in several baths of xylene and a thin layer of polystyrene mountant was applied, followed by a glass coverslip.

### Bioinformatic Analysis

The RNAseq data from GDC TCGA lung adenocarcinoma (LUAD) patient cohort were collected for analysis (tcga‐data.nci.nih.gov/). The data were processed under R environment (version 4.1.2, https://www.r‐project.org/). The samples were divided into two groups, tumor tissues or adjacent normal tissues. The UPS‐related gene set containing 676 UPS‐correlated genes according to a previous study and conducted differential expression genes (DEGs) analysis based on this data set were collected, using the DESeq2 package in R (available at https://bioconductor.org/packages/3.16/bioc/html/DESeq2.html).^[^ ^29^ ^]^ Gene ontology (GO) enrichment analysis was further conducted to define the correlation of these DEGs with UPS system. The “GOplot” package was employed to visualize the enrichment results.^[^ ^30^ ^]^ Univariate Cox regression analysis was performed to identify prognosis‐related UPS genes. And among these genes, Lasso regression analysis was further utilized to define independent prognostic genes. Hence, a prognostic model was constructed, and patients were divided into two groups of low risk or high risk according to the expression of these genes. Then survival analysis was performed by Kaplan‐Meier method. For the RNAseq analysis in vitro, normal A549 and starvation‐adapted A549 cells were collected and total RNA was extracted using Qiagen RNeasy Mini Kit (Qiagen, 74 104). DEGs analysis was performed and heatmap plot was generated using pheatmap package. GSEA analysis was performed to validate the correlation of DEGs and UPS activity in GSEA software (version 4.0.1).

### Statistical Analysis

GraphPad Prism 8.0 software (GraphPad Software, Inc) was used for statistical data analysis. To compare the differences between two groups, two‐tailed unpaired Student's t‐test was used. To analyze and compare the differences among multiple‐group means, one‐way analysis of variance (ANOVA) with Tukey's multiple comparison test was applied. For anti‐cancer effect in vivo, two‐way ANOVA with Tukey's multiple comparison analysis was employed. Values of P < 0.05 were considered significant. Results were represented as means ± SD. ^*^ *p* <0.05, ^**^ *p* <0.01, ^***^ *p* <0.001, ^****^ *p* <0.0001.

## Conflict of Interest

The authors declare no conflict of interest.

## Supporting information

Supporting Information

## Data Availability

The data that support the findings of this study are available from the corresponding author upon reasonable request.
