# Supplementary material for: Amino Acid‐Starved Cancer Cells Utilize Macropinocytosis and Ubiquitin‐Proteasome System for Nutrient Acquisition
Source: Adv Sci (Weinh). 2023 Nov 20;11(1):2304791. doi: 10.1002/advs.202304791 (PMC10767443; doi:10.1002/advs.202304791)
Supplement: Supplementary file 1 — Supporting Information [file ADVS-11-2304791-s001.pdf]

ADVANCED SCIENCE
Open Access

Supporting Information

for *Adv. Sci.*, DOI 10.1002/advs.202304791

Amino Acid-Starved Cancer Cells Utilize Macropinocytosis and Ubiquitin-Proteasome System for Nutrient Acquisition

*Tianyi Wang, Yaming Zhang, Yuwei Liu, Yi Huang and Weiping Wang**

Supporting Information

Amino acid-starved cancer cells utilize macropinocytosis and ubiquitin-proteasome system for nutrient acquisition

Tianyi Wang[1,2,3†], Yaming Zhang[1,2,3†], Yuwei Liu[1,2,3], Yi Huang[1,2,3], Weiping Wang[1,2,3,]*

[1]State Key Laboratory of Pharmaceutical Biotechnology, The University of Hong Kong, Hong Kong, China

[2]Department of Pharmacology and Pharmacy, Li Ka Shing Faculty of Medicine, The University of Hong Kong, Hong Kong, China

[3]Dr. Li Dak-Sum Research Centre, The University of Hong Kong, Hong Kong, China

†These authors contributed equally to this work

*Corresponding author email: wangwp@hku.hk

Gene	HR	lower 95%CI	upper 95%CI	pvalue
UBE2T	1.211	1.058	1.386	0.006
CDC20	1.218	1.087	1.365	0.001
UHRF1	1.331	1.121	1.580	0.001
UBE2C	1.163	1.056	1.281	0.002
CDCA3	1.322	1.107	1.580	0.002
PRC1	1.384	1.188	1.613	0.000
AURKA	1.270	1.104	1.462	0.001
CCNF	1.261	1.023	1.556	0.030
RNF144B	0.816	0.675	0.987	0.036
DTL	1.445	1.202	1.738	0.000
TRIM59	1.534	1.139	2.066	0.005
KAT2B	0.721	0.564	0.921	0.009
UBE2S	1.318	1.133	1.533	0.000
BRCA1	1.299	1.054	1.600	0.014
WDR76	1.340	1.080	1.662	0.008
TRIM2	0.829	0.717	0.959	0.012
NEDD4L	0.825	0.683	0.997	0.047
KLHL10	0.008	0.000	0.854	0.043
RNF180	0.646	0.486	0.858	0.003
TRIM6	1.451	1.200	1.754	0.000
USP44	0.740	0.571	0.958	0.023
TRIM40	1.684	1.079	2.628	0.022
TRIML2	2.075	1.227	3.506	0.006

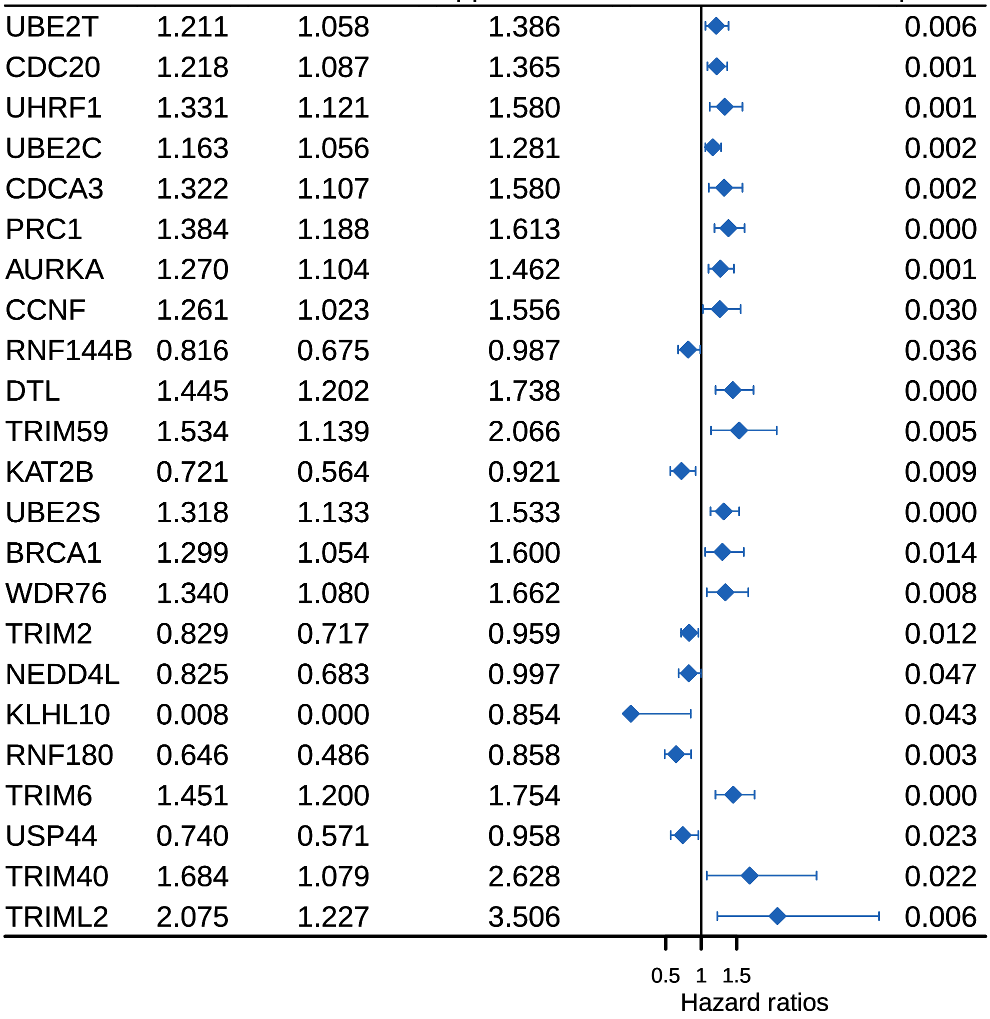

Figure S1. 23 UPS genes that may influence prognosis in lung adenocarcinoma patients analyzed by univariate Cox regression.

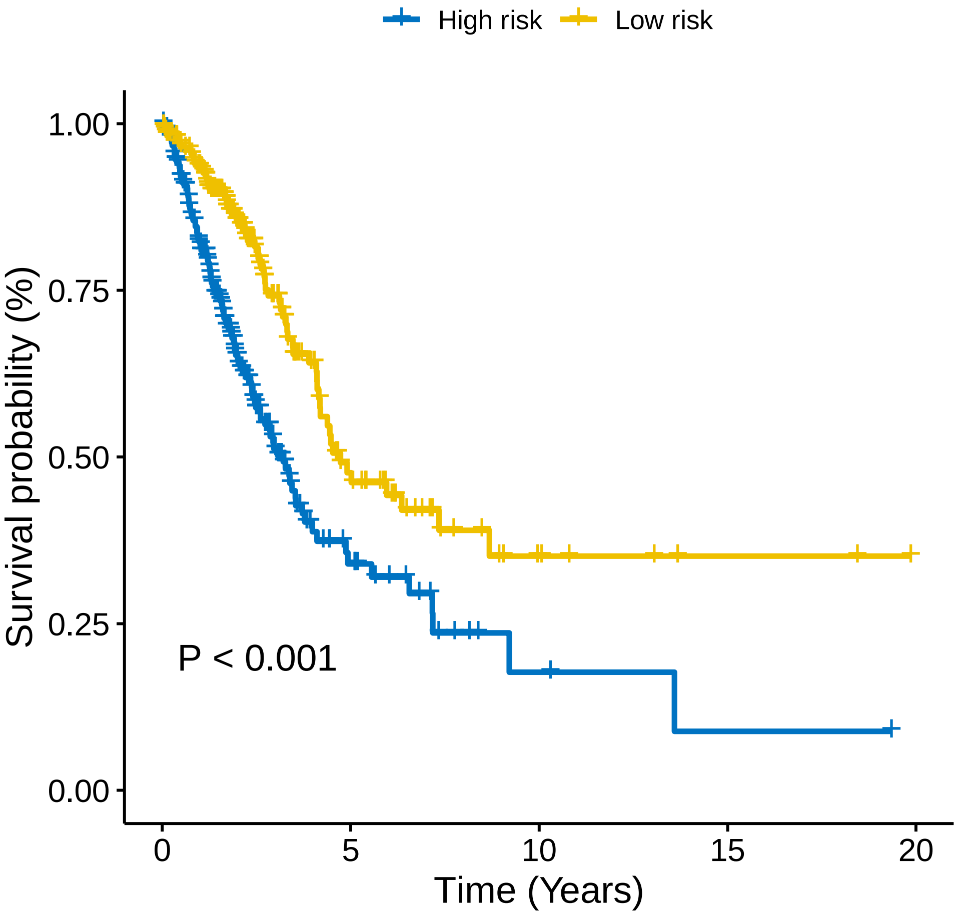

Figure S2. Expression of PRC1, TRIM6, and TRIML2 may influence the survival of lung adenocarcinoma patients as independent risk factors. n = 248 for the group with high risk, n = 249 for the group with low risk.

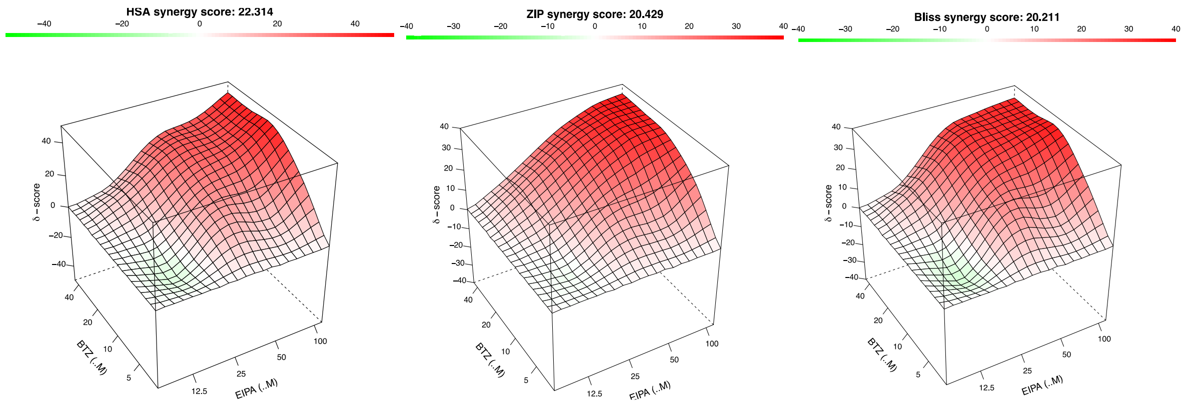

Figure S3. Mean synergy score of EIPA and BTZ combination calculated by SynergyFinder website.

Three calculating models were applied, including HSA, Bliss, and ZIP models.

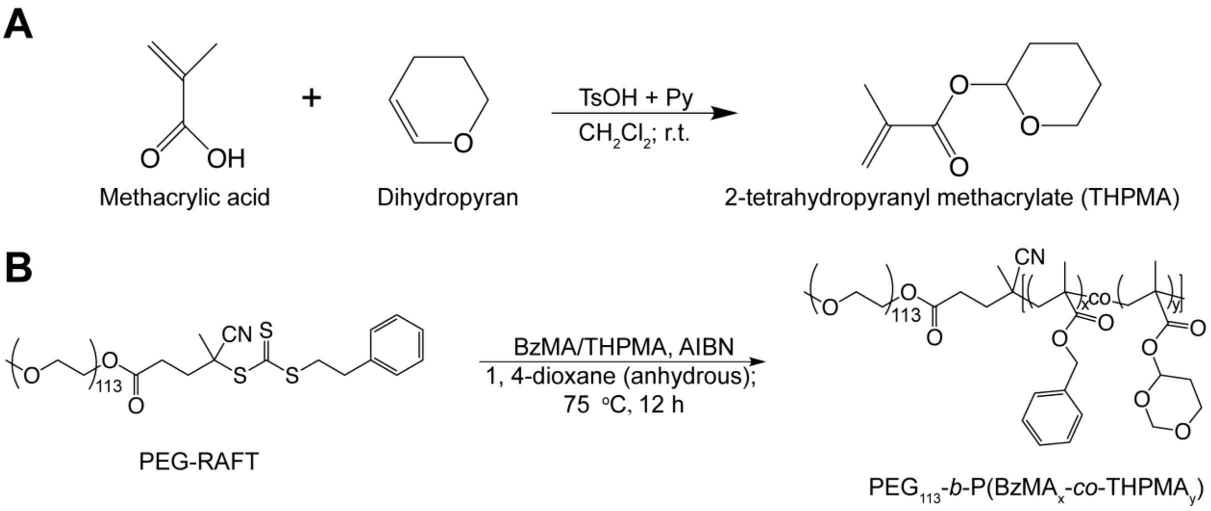

Figure S4. Synthetic routes of the block copolymers.

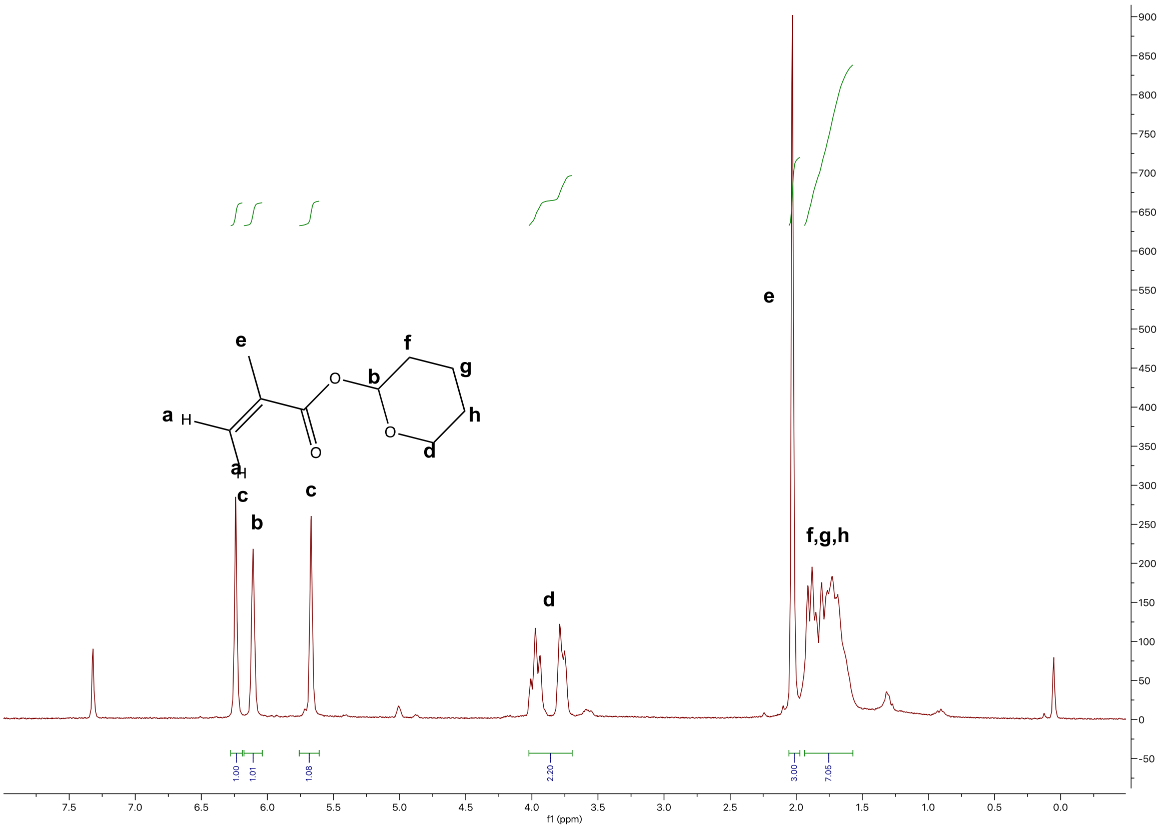

Figure S5. ^{1}H-NMR spectrum of THPMA

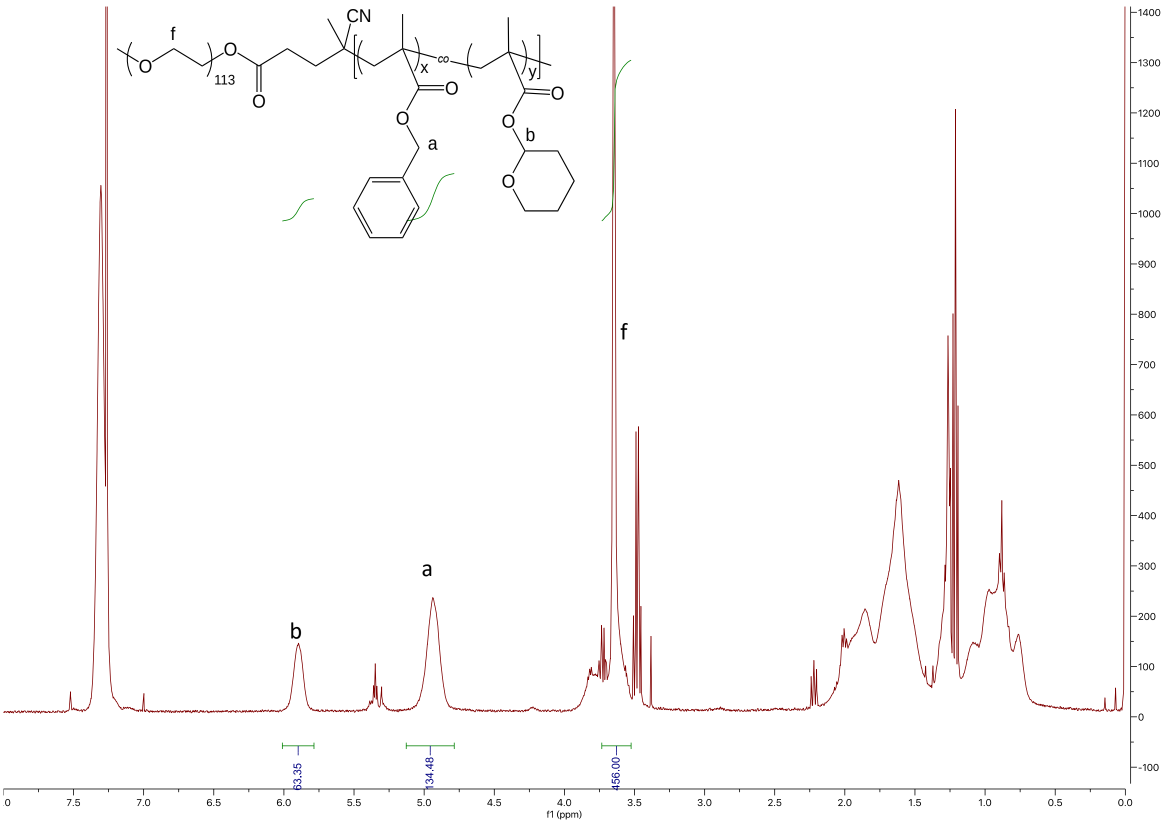

Figure S6. ^{1}H-NMR spectrum of PEG_{113}-*b*-P($BzMA_{68}$-*co*-$THPMA_{63}$).

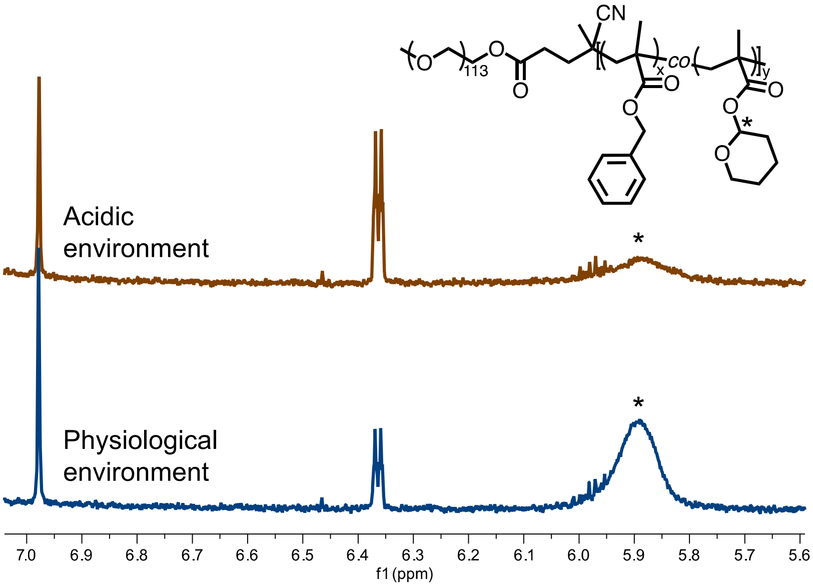

Figure S7. pH-responsive cleavage of PEG-*b*-P(BzMA-*co*-THPMA) under acidic environment for 24 h.

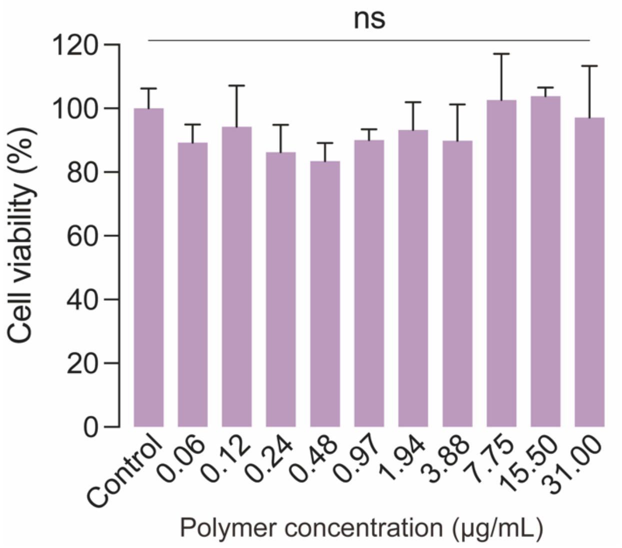

Figure S8. Cell viability analysis of HUVEC cells treated with different concentration of polymersomes (without drug loading) for 24 h.

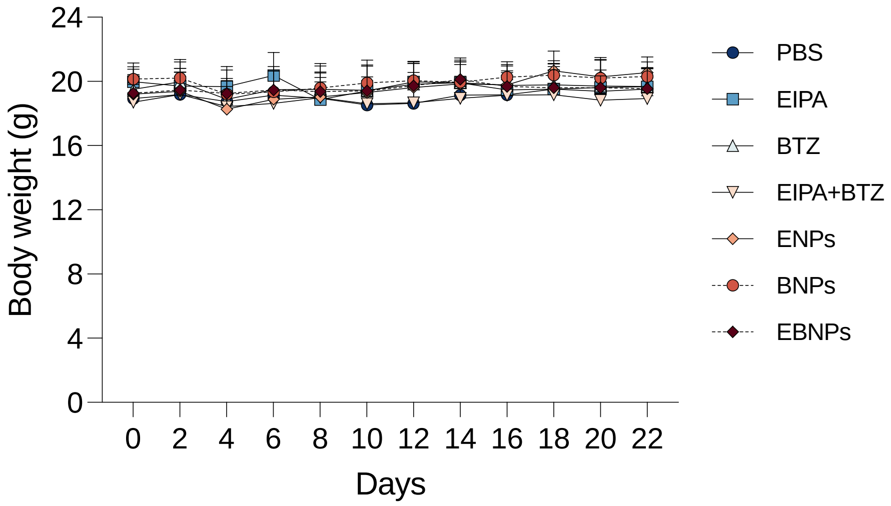

Figure S9. Body weight profiles of the tumor-bearing mice receiving different treatments.

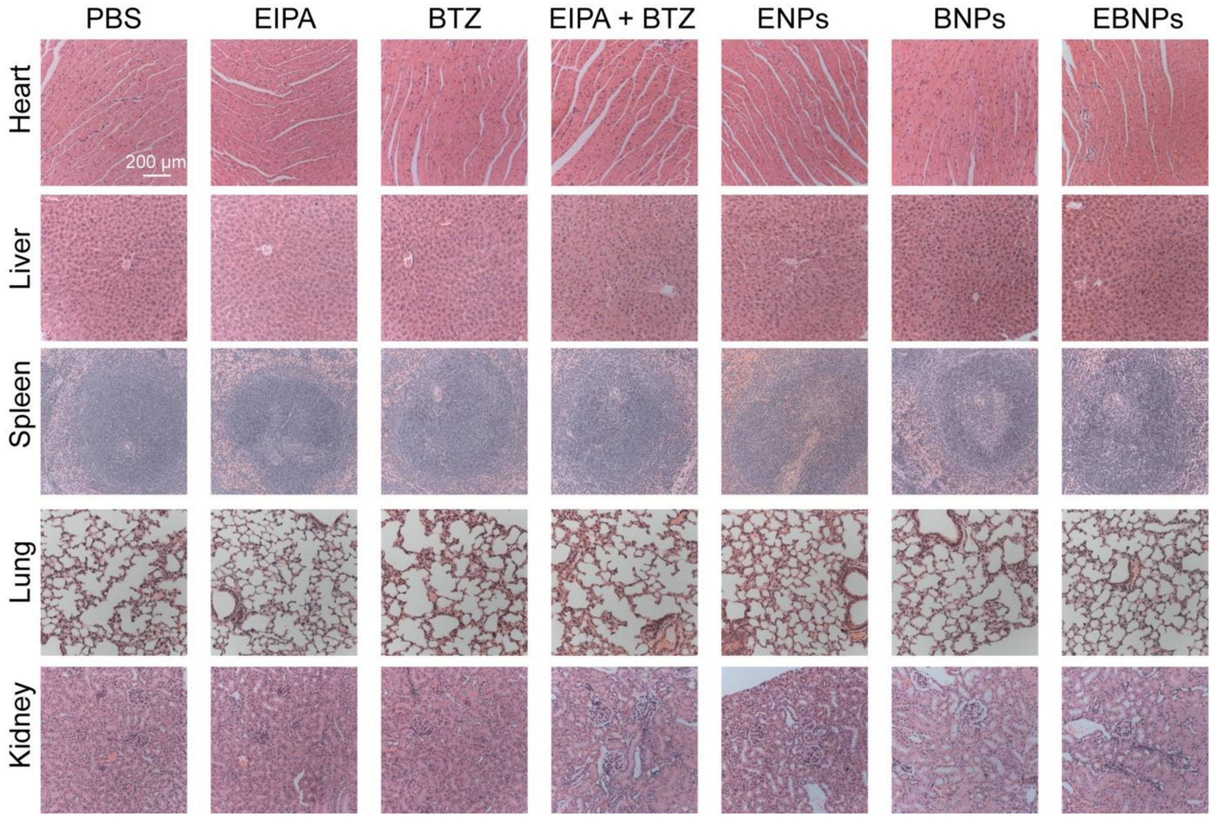

Figure S10. H&E staining profile of major organs from the tumor-bearing mice, including heart, liver, spleen, lung, and kidney, at the end of different treatments. Scale bar: 200 µm
